# What Family Circumstances, During COVID-19, Impact on Parental Mental Health in an Inner City Community in London?

**DOI:** 10.3389/fpsyt.2021.725823

**Published:** 2021-12-16

**Authors:** Lydia Whitaker, Claire Cameron, Hanan Hauari, Katie Hollingworth, Margaret O'Brien

**Affiliations:** Thomas Coram Research Unit, University College London, London, United Kingdom

**Keywords:** mental health, financial insecurity, poverty, food poverty, child mental health, inner city

## Abstract

The introduction of lockdown due to a public health emergency in March 2020 marked the beginning of substantial changes to daily life for all families with young children. Here we report the experience of families from London Borough of Tower Hamlets with high rates of poverty and ethnic and linguistic diversity. This inner city community, like communities worldwide, has experienced a reduction or closure in access to education, support services, and in some cases, a change in or loss of income, job, and food security. Using quantitative survey items (*N* = 992), we examined what differences in family circumstances, for mothers and fathers of young children aged 0–5 living in Tower Hamlets, during March 2020 to November 2020, were associated with their mental health status. We measure parental mental health using symptoms of depression (self-report: Patient Health Questionnaire depression scale: PHQ-8), symptoms of anxiety levels (self-report: General Anxiety Disorder: GAD-7), and perceptions of direct loneliness. We find parental mental health difficulties are associated with low material assets (financial security, food security, and children having access to outside space), familial assets (parents time for themselves and parent status: lone vs. cohabiting), and community assets (receiving support from friends and family outside the household). South Asian parents and fathers across ethnicities were significantly more likely to experience mental health difficulties, once all other predictors were accounted for. These contributing factors should be considered for future pandemics, where restrictions on people's lives are put in place, and speak to the importance of reducing financial insecurity and food insecurity as a means of improving the mental health of parents.

## Introduction

Tower Hamlets, an inner city borough in London, has a unique profile with a broad spectrum of income and health inequalities. Borough citizens range from the wealthy to those living in poverty. Prior to the COVID-19 pandemic, Tower Hamlets was already considered high risk at a national level (1) with a deprivation score of 35.7, in contrast to England‘s score of 21.8. Furthermore, more children in Tower Hamlets were from low income households (30%) than London (19%) and England (17%) (1). This community includes people from a multitude of ethnic backgrounds; for the purposes of this paper we focus on two clusters who made up the majority of our sample, White British/Irish and South Asian (Bangladesh, India, and Pakistan). In Tower Hamlets, residents from White backgrounds total 34% while Asian residents total 48% of the population (2).

The Families in Tower Hamlets project is a mixed methods longitudinal study aiming to examine how financial, social, and health aspects of families with children under five and pregnant women are influenced during the pandemic using an assets-based approach (3). The assets-based approach explores assets and resources that aid or hinder children's, parents', families', and community development. Assets are present at every level of a child's life; individual, familial, community, and institutional, and act at an individual level, for example manifesting in one's beliefs or motivations (4). In contrast, resources are external factors, such as support from friends and family in the child's pursuits or aims. Both of these act as protective factors in a child's development (4). Here we explore material (financial security, income, food security, and children's access to outside space), familial (partner type: cohabiting vs. lone, parental time for self), community (support from friends and family outside the household), and adequate housing assets as potential protective factors for parental mental health. We examine maternal and paternal mental health, excluding adults that were soon to be parents (pregnant).

Working in partnership with borough health, care, and education service providers we seek to aid adaptation of service provision in light of changed needs that may have arisen since the pandemic. The design of this project mirrors that of a survey of mothers as part of the Born in Bradford family of studies (5), under the auspices of ActEarly (6). ActEarly is a location-based city collaboratory aiming to harness local authority expertise, academic research, and community engagement to improve the health and opportunities for children living in two contrasting areas with ethnically diverse populations and high levels of child poverty; Bradford, West Yorkshire and Tower Hamlets, London (https://actearly.org.uk/).

## Mental Health

We examine parental mental health, defined as an individual's ability to manage life and work stresses, be able to understand and comprehend their own abilities, and be a contributing part to their community (7). Over half of adults with mental health difficulties are parents [57% of men and 68% of women: (8)]. Multiple factors, before COVID-19, were associated with parental mental health difficulties. Financial insecurity (9, 10) and low income (11, 12) are two such factors. Food insecurity (13) and overcrowding (14) are other factors associated with poor physical and mental health, and are greater in financially worse off households (14) and households with children (13).

Parents in Tower Hamlets, like the rest of the UK, experienced competing and multiple demands on their time, resources, and mental capacity during lockdowns that began in March 2020 and went on, in varying degrees of intensity to the time of writing. From March 2020, lockdowns restricted movement and social mixing, schools and early childhood education settings were closed to most children as well as a reduction to health and social care provision, and restrictions to daily activities (15). The lockdown eased from June 1 onwards with the cautious reopening of workplaces, schools, and ECEC services as well as easing of restrictions on daily activities and mobility. Social mixing restrictions included associations with up to six people (including your household) in September and in October 2020, residents were restricted to socializing only within the household or support bubble. While working from home was mandated by government, where possible, front line services continued and many Tower Hamlet residents would have had to keep working [60% of the Tower Hamlets sample were employed at the time of the survey: (16)]. The lockdown restrictions meant that for parents, financial insecurity and work stresses became more prominent and their support network reduced or changed (17). The social isolation experienced through restricted access to outdoor space, friends, and family and increased time helping children with home learning meant for many adults an increased likelihood of mental health difficulties (18).

For a group of adults whose depression and anxiety rates were higher than national levels [Tower Hamlets: 16.1%, National figures: 13.7%: (19)] before COVID-19, coupled with changes in life stresses and support networks, the financial and work stresses associated with COVID-19 led to increased pressures on families in Tower Hamlets.

Increases in poor mental health at the onset of COVID-19 in England were found across men and women, with more pronounced increases for women (20). South Asian (India, Pakistan, and Bangladesh) and White men experienced increases in poor mental health, with greater prevalence for Pakistani and Bangladeshi men compared to White men (21). Asian adults reported greater feelings of loneliness, compared to White adults (21). While there are no national prevalence data on mental health patterns for parents during COVID-19, a recent study has found a temporary mental health decline for both mothers and fathers of primary school aged children during school closures, with a greater impact for mothers (22).

Previous crises in high income countries, which have resulted in governments restricting residents' movements, have had a detrimental effect on parental mental health (18). There is increasing concern over the impact of lockdown and COVID-19 on the mental health of parents and their children (23–25). With poor parental mental health being associated with poor child mental health (26–28). This is of great relevance in Tower Hamlets, in which 11% of children (5–16 years) had mental health disorders (1).

When we consider the above evidence of triggers associated with parental mental health, in the context of Tower Hamlets, where triggers of poor mental health, such as low income are high, and mental health difficulties are higher than national levels, we need to understand what facilitates and prevents poor parental mental health.

## Possible Facilitators and Preventers of Parental Mental Health During COVID-19

### Material Assets (Financial Security and Income)

Marked changes in financial insecurity were seen in the UK during 2020, with increases in unemployment (29) and uptake of Universal Credit claims (30). As in pre-COVID-19 times (31), parents and adults from low income households are more likely to experience mental health problems during COVID-19 compared to those from higher income households (32). Prior to COVID-19, financial insecurity and low income have been associated with mental health difficulties (10, 17). Financial insecurity (loss of job/change in financial circumstances) for parents (9) has been associated with heightened stress (33) and often propels depression, anxiety, and loneliness [adults: (34); parents: (17); loneliness: (35)]. Increase in stress and poor mental health are associated with heightened substance use, aggression, and abuse (36, 37) in the home (38). There is evidence of these negative aspects of human life increasing over lockdown [domestic violence/child abuse: (39), substance abuse (40)].

Increases in parental depression and anxiety since COVID-19 are related to financial insecurity (17, 41) further exacerbated by income level and gender differences (17). Those who already experienced financial difficulties prior to COVID-19 have greater mental health difficulties with the additional financial strain experienced during COVID-19 (31). Sustained mental health difficulties in the UK are more common in adults from ethnic minorities and those experiencing financial insecurities (42). We consider these material asset facilitators of poor mental health within the context of Tower Hamlets, a borough in which child poverty is the highest in England once costs of housing are taken into consideration (43), and Bangladeshi families in particular are at higher risk of poverty and employment precarity (44).

### Adequate Food

Food insecurity has increased since COVID-19 (45) perhaps due to the increase in waiting time for Universal Credit and school meal vouchers as well as job losses. Food insecurity is of high concern in Tower Hamlets (46); the borough launched an emergency food programme in Spring 2020, which diverted large scale donations of food to over 30 community organizations for further distribution to families as a protection against hunger (Vafai et al., p.c). Food insecurity is associated with mental health difficulties during COVID-19 (47) and an increase in depression during COVID-19 (41).

### Adequate Housing

We report that 16.8% of Tower Hamlets parents lived in households with a single bedroom (16) whereas Census (48) data found that Tower Hamlets residents live in households with 2.1 bedrooms per household and 2.5 persons per household. This is in contrast to London (2.0 persons per household) and Bradford (2.6 persons per household) [Housing projections database (49)].

People living in poverty are more likely to live in overcrowded housing (50, 51).^1^ The additional strain of lockdown restrictions on leaving home, as well as lack of access to outside space (e.g., a garden) has been hailed as a catalyst for health concerns in this group of people during COVID-19 (50). Overcrowding is associated with mental health difficulties, especially in lone parent households (14). As yet, the specific relationship between overcrowding and mental health during COVID-19 is unknown. We will examine the role adequate housing plays in parental mental health.

### Relationships and Support: Community and Familial Assets

We were also interested in the role social relationships play in the mental health of parents with children below 5 years of age. In line with lockdown restrictions (October-November 2020), households were advised not to see their non-household (friends and family outside the household) support network or were limited to six people in a social interaction. These restrictions no doubt left many parents feeling socially isolated, and national (35, 52) as well as inner city evidence (5) have found that social isolation and loneliness are associated with depression and anxiety during COVID-19. In light of the limited access to a non-household support network lockdown brought, we wanted to examine community support influence on parental mental health. We deemed support to be an additional means to examine social isolation and mental health in parents (35, 52). We build on Born in Bradford's findings of the importance of this community factor on parental mental health in our own sample (5).

With these restrictions, on outside activity, as well as overcrowding inside, the quality of a parent's relationship with their spouse becomes even more important. The strain of lockdown has been documented with increased incidents of domestic abuse (36, 37) and incidents of poor relationship quality with their partners and children (53). Yet there is evidence that the strains of lockdown have also brought parents and family relationships with partners and children closer together (16, 53, 54). We consider the community and familial influences on mental health in cohabiting and lone parents as well as access to support from friends and family (outside the household) in Tower Hamlets.

### Time for One's Self

The competing pressures on parents' lives and lack of separation between work and home, as a result of lockdown restrictions, means that parents have less time to themselves (17). More time doing such leisure activities as exercising and gardening facilitates improvements in mental health during COVID-19 (55). We consider the role of changes in parental time for one's self, since before COVID-19, as familial assets, on parental mental health (56).

### The Present Study

The majority of previous studies mentioned above, with the exception of Born in Bradford (5, 57), rely on respondents from predominantly White backgrounds from across England and the United Kingdom. Our sample, indicative of inner city Tower Hamlets, is ethnically diverse. The current study is uniquely placed to shed light on the mental health of inner city parents, with substantial poverty prior to COVID-19. With the effects of COVID-19 exacerbating pre-existing triggers of poor mental health [(17, 34)], greater prevalence in people from ethnic minorities (58), and those financially worse off (59), this study is of importance at a local (Tower Hamlets council, Docklands Outreach or Step Forward) and national level (ActEarly, Department of Health and Social Care).

Our aims are 2-fold: (i) to examine any changes in material assets (financial security compared to before COVID-19) or familial assets (changes in time for one's self since before COVID-19) in parents lives, that may influence their mental health; (ii) to examine the possible facilitators and exacerbators of poor mental health of parents in Tower Hamlets, looking at financial, community, adequate housing, and familial assets. Below we draw on the possible associations between familial assets (relationship with partner, parent status: lone/ cohabiting), material assets (income, financial insecurity, food insecurity, and child's access to outdoor space), adequate housing assets and community assets (giving and receiving support from outside the household), and mental health.

## Materials and Methods

The Families in Tower Hamlets project includes three strands. Firstly, a repeat community-based online survey, in which this paper focuses on the first wave, secondly, a repeat in-depth qualitative household interview panel, and thirdly mapping of changes to borough services for families and children under five.

In this paper we focus on Wave 1 of the survey and specifically parents from White British/Irish and South Asian (Bangladesh, India, and Pakistan) backgrounds and exclude respondents who are pregnant with no children under 5 years of age. For details on the wider sample see Cameron et al. (16).

Recruitment of a community sample was conducted by our local authority partners in the London Borough of Tower Hamlets. Strategies adopted to recruit participants included a borough-wide social media campaign, entries in residents' magazines and newsletters, as well as invitations to participate through the borough's child-facing services (health visiting teams, early years and family support), and civil society organizations (faith-based organizations and community centers). Parents received a £10 shopping voucher for their contribution to the project.

### Sample

Our sample consisted of parents of children under 5 years old who were residents in Tower Hamlets. Mothers made up the majority of the sample (71%) in the current paper. A relatively equal split of ethnic groups was found with 44% being from White British/Irish backgrounds and 56% from South Asian (Bangladesh, India, and Pakistan) backgrounds (see Table 1).

**Table 1:** Description of Tower Hamlets sample by ethnicity and parent gender.

	**Mothers**		**Fathers**		**Total**	
	***N***	**%**	***N***	**%**	***N***	**%**
White British/Irish	191	39.7	109	55.9	300	44.4
South Asian	290	60.3	86	44.1	376	55.6
Total	481	100	195	100	676	100

### Procedure

Using opt-in community-based recruitment, respondents were asked about themselves and their family's lives pre-COVID-19 and during the pandemic (July 2020-November 2020) using an online survey tool (Qualtrics). Areas relating to financial security, family structure, adequate housing, food security, outdoor space, and quality of relationship are discussed in this paper.

For an overview of the topics covered in the survey and the wider sample see Cameron et al. (16). Wave 2 (February-May 2021) of the Families Tower Hamlets project will examine changes in depression, anxiety, and loneliness during COVID-19.

UCL Institute of Education Research Ethics Committee and the NHS Health Research Authority awarded ethical approval.

## Results and Discussion

We summarize findings from Table 2 four multiple hierarchical linear regressions (60). The results of the hierarchical regressions for depression (Table 3), anxiety (Table 4), loneliness (Table 5), and anxiety in mothers (Table 6) are outlined below respectively.

**Table 2:** Correlation coefficients between mental health and predictors.

			**1**	**2**	**3**	**4**	**5**	**6**	**7**	**8**	**9**	**10**	**11**	**12**	**13**	**14**	**15**	**16**
			**Lone-liness**	**Depression**	**Anxiety**	**Ethnicity**	**Sex**	**Income**	**Over-crowding**	**Food insecurity**	**Leisure time**	**Quality relation-ship**	**Receive support**	**Parental status (lone vs. cohabiting)**	**Give support**	**Financial insecurity**	**Children have access to outdoor space**	**Financial insecurity compared to before March 2020**
1	Loneliness		1	−0.477^**^	−0.438^**^	−0.018	−0.014	0.208^**^	0.032	−0.278^**^	0.097^**^	−0.024	0.001	0.194^**^	−0.061	0.248^**^	0.058	0.038
		*N*		709	706	541	723	622	719	720	711	622	721	705	695	713	368	690
2	Depression			1	0.802^**^	0.109^**^	0.002	−0.234^**^	0.049	0.302^**^	−0.210^**^	0.010	0.099^**^	−0.152^**^	0.086^*^	−0.378^**^	−0.153^**^	−0.043
		*N*			778	592	788	672	783	784	742	663	781	769	746	771	397	745
3	Anxiety				1	0.070	0.026	−0.206^**^	0.047	0.310^**^	−0.199^**^	−0.008	0.099^**^	−0.109^**^	0.085^*^	−0.368^**^	−0.095	−0.081^*^
		*N*				590	785	671	780	780	736	661	777	766	743	767	395	742
4	Ethnicity					1	−0.148^**^	−0.415^**^	0.354^**^	0.095^*^	−0.248^**^	0.134^**^	−0.013	−0.065	−0.099^*^	−0.346^**^	−0.090	0.159^**^
		*N*					676	543	654	617	566	533	603	652	577	604	324	583
5	Sex						1	0.005	−0.068^*^	−0.059	0.283^**^	−0.066	−0.076^*^	0.236^**^	0.032	−0.014	0.013	−0.068
		*N*						716	864	820	753	680	802	858	766	805	434	780
6	Income							1	−0.173^**^	−0.235^**^	−0.015	−0.023	−0.054	0.296^**^	−0.011	0.526^**^	0.038	0.052
		*N*							711	698	644	588	685	702	655	694	374	675
7	Overcrowding								1	0.068	−0.154^**^	0.029	0.002	0.043	−0.020	−0.141^**^	−0.079	0.031
		*N*								814	749	676	798	841	762	799	432	775
8	Food insecurity									1	−0.126^**^	−0.010	0.001	−0.111^**^	0.025	−0.229^**^	−0.066	−0.035
		*N*									748	675	796	800	761	802	410	777
9	Leisure time										1	−0.061	−0.084^*^	0.102^**^	−0.012	0.124^**^	0.062	−0.108^**^
		*N*										648	752	733	723	737	379	713
10	Quality relationship											1	−0.003	−0.168^**^	−0.028	−0.065	0.029	0.051
		*N*											678	666	655	662	343	646
11	Receive support												1	−0.059	0.272^**^	−0.095^**^	0.010	−0.028
		*N*												781	764	782	397	759
12	Parental status (lone vs. cohabiting)													1	0.007	0.215^**^	0.109^*^	−0.046
		*N*													746	785	425	760
13	Give support														1^**^	0.001	0.040	−0.046
		*N*														748	383	725
14	Financial insecurity															1	0.070	0.078^*^
		*N*															404	766
15	Children have access to outdoor space																1	0.024
		*N*																388
16	Pre–COVID−19 Financial insecurity																	1
		*N*																

*Levels of significance:*

* *p, 0.05;*

** *p, 0.01;*

*** *p, 0.001*

**Table 3:** Hierarchical regression analyses predicting symptoms of depression.

**Depression**		***SE b***	**β**	***t***	***N***
Step 1	Parental gender	0.17	0.07	1.13	241
	Ethnicity	0.15	−0.1	−1.64	241
	Current Financial insecurity	0.15	−0.48^***^	−8.06	241
			*R*^2^ = 0.23		
Step 2	Parental gender	0.17	0.12^*^	2.13	241
	Ethnicity	0.16	−0.13^*^	−2.02	241
	Current Financial insecurity	0.17	−0.31^***^	−4.34	241
	Parental status (lone vs. cohabiting)	0.21	0.01	0.09	241
	Give support	0.15	0.1	1.73	241
	Received support	0.15	−0.04	−0.69	241
	Overcrowding	0.19	−0.04	−0.59	241
	Food insecurity	0.24	0.23^***^	3.82	241
	Time for one's self	0.08	−0.11	−1.91	241
	Income: Low income (< £20,799), Mid-income (£20,800–£51,999), High income (£52,000 and above)	0.11	−0.13	−1.82	241
	Child has access to outdoor space	0.17	−0.13^*^	−2.33	241
		Δ*R*^2^ = 0.13		

*Levels of significance:*

* *p, 0.05;*

** *p, 0.01;*

*** *p, 0.001*.

**Table 4:** Hierarchical regression analyses predicting symptoms of anxiety.

		***SE b***	**β**	***t***	***N***
**Anxiety**					
Step 1	Parental gender	0.091	0.07	1.65	449
	Ethnicity	0.089	−0.09^*^	−2.1	449
	Current Financial insecurity	0.089	−0.37^***^	−8.36	449
	Food insecurity	0.145	0.25^***^	5.94	449
	Time for one's self	0.05	0.19^***^	−4.3	449
			*R*^2^ = 0.30		
Step 2	Parental gender	0.093	0.09^*^	2.05	449
	Ethnicity	0.1	−0.079	−1.6	449
	Current Financial insecurity	0.101	−0.35^***^	−6.9	449
	Food insecurity	0.147	0.26^***^	5.99	449
	Time for one's self	0.052	−0.17^***^	−3.92	449
	Parental status (lone vs. cohabiting)	0.131	−0.06	−1.5	449
	Give support	0.085	0.03	0.67	449
	Received support	0.085	0.10^*^	2.37	449
	Overcrowding	0.11	−0.01	−0.17	449
	Income: Low income (< £20,799), Mid-income (£20,800–£51,999), High income (£52,000 and above)	0.068	0.02	0.36	449
		Δ*R*^2^ = 0.014		

*Levels of significance:*

* *p, 0.05;*

** *p, 0.01;*

*** *p, 0.001*.

**Table 5:** Hierarchical regression analyses predicting direct loneliness.

		***SE b***	**β**	***t***	***N***
**Loneliness**					
Step 1	Parental gender	0.04	−0.046	−1.03	455
	Ethnicity	0.039	0.065	1.42	455
	Current Financial insecurity	0.039	0.17^***^	3.66	455
	Parental status (lone vs. cohabiting)	0.056	0.19^***^	4.27	455
	Food insecurity	0.062	−0.29^***^	−6.57	455
			*R*^2^ = 0.20		
Step 2	Parental gender	0.041	−0.064	−1.38	455
	Ethnicity	0.041	0.096	1.97	455
	Current Financial insecurity	0.044	0.14^*^	2.58	455
	Parental status (lone vs. cohabiting)	0.057	0.18^***^	4.02	455
	Food insecurity	0.063	−0.28^***^	−6.25	455
	Time for one's self e	0.023	0.079	1.67	455
	Income: Low income (< £20,799), Mid–income (£20,800–£51,999), High income (£52,000 and above)	0.031	0.067	1.22	455
		Δ*R*^2^ = 0.01		

*Levels of significance:*

* *p, 0.05;*

** *p, 0.01;*

*** *p, 0.001*.

**Table 6:** Hierarchical regression analyses predicting anxiety in mothers.

		***SE b***	**β**	***t***	***N***
**Anxiety in mothers**					
Step 1					
	Ethnicity	0.106	0.05	1.01	371
			*R*^2^ = 0.003		
Step 2					
	Ethnicity	0.104	0.01	0.18	371
	Time for one's self	0.065	−0.22^***^	−4.27	371
	Parental status (lone vs. cohabiting)	0.131	−0.17^**^	−3.44	371
		Δ*R*^2^ = 0.08		

*Levels of significance:*

* *p, 0.05;*

** *p, 0.01;*

*** *p, 0.001*.

### Data Cleaning and Analyses

Mental health data were collected using standardized measures of depression [Patient Health Questionnaire depression scale: PHQ-8, (61)] and anxiety [General anxiety disorder: GAD-7, (62)]. The PHQ-8 is an 8-item instrument with a 4-item scale (not at all, score = 0, 1 or 2 days, score = 1, more than half the days, score = 2, nearly every day, score = 3). A score of 0–4 = no depressive symptoms, 5–9 = mild depression, 10–14 = moderate depression, 15–19 = moderately severe depression, and 20–24 = severe depression. The GAD-7 is a 7-item instrument with a 4-item scale (not at all, score = 0, 1 or 2 days, score = 1, more than half the days, score = 2, nearly every day, score = 3). A score of 5 = mild anxiety, 10 = moderate anxiety, and 15 or more = severe anxiety. The PHQ-8 and GAD-7 have been used across populations and paradigms (63, 64) to measure depression and anxiety.

We define loneliness as a perception of being isolated and alone. Direct loneliness was collapsed as follows; not feeling lonely (“none or almost none of the time” or “some of the time”) and feeling lonely (“most of the time” or “all or almost all of the time”). For a discussion on using a multiple linear regression using dichotomous outcome variables see Battey et al. (65). We include a direct measure of loneliness, that is feelings of loneliness in the present, in line with ONS guidelines (66). The loneliness instrument was an abridged version of that used in ONS (67).

Income was collapsed into tertiles (low income: < £20,799, mid-income: £20,800–£51,999, high income: £52,000 and above). Overcrowding was calculated by dividing number of bedrooms in the household by number of household members. Gender of parent was defined as follows: mother = female with a child under 5 hence forth referred to as mothers, father = male with a child under 5 hence forth referred to as fathers.

The following variables were collapsed as part of regression analyses. Food insecurity: secure (“sometimes true” or “never true” that food did not last) and insecure (“often true” that food did not last). Financially insecurity: secure (“living comfortably” or “doing alright”) and insecure (“just about getting by” or “finding it quite difficult” or “finding it very difficult”). Financial insecurity compared to pre-COVID-19 secure (“better off” or “about the same”) and insecure (“worse off”). **Change since COVID-19:** time for one's self **prior to COVID-19**; less time (“much less time” or “slightly less time”), just as much time (“just as much time”), or more time (“slightly more time” or “much more time”) [in line with (56)].

### Selection for Final Regression Models

Possible predictors of mental health: parental gender, ethnicity, financial insecurity compared to pre-COVID-19, current financial insecurity, income, children's access to outdoor space, food insecurity, quality of relationship, parent type (lone /cohabiting), support (received and given) from/to friends and family outside household, time for one's self prior to COVID-19, and overcrowding were included in correlations. Any non-significant predictors were excluded from regression models (quality of relationship, financial insecurity compared to pre-COVID-19) with the exception of predefined predictors which we deemed to have sufficient empirical importance to include. A said predictor was overcrowding. Materials for variables financial insecurity compared to pre-COVID-19, current financial insecurity, income, children's access to outdoor space, food insecurity, quality of relationship, parent type (lone /cohabiting), support (received and given) from/to friends and family outside household, and time for one's self prior to COVID-19 were taken from previous surveys that assessed reliability and validity of instruments (56)^2^.

### Variables Examining Change

Financial insecurity compared to pre-COVID-19 was not significantly correlated with mental health (depression, anxiety, and loneliness) and was excluded from regression analyses. Time for one's self **prior to COVID-19** was included in regression models.

### Parental Mental Health During COVID-19

We explored whether parental gender, ethnicity, financial insecurity, parent status (lone parent vs. cohabiting), support (giving and receiving) to/from friends and/or family outside of the household, overcrowding (number of bedrooms/number of household members), food insecurity, time for one's self prior to COVID-19, income, predicted self-reported depressive symptoms, symptoms of anxiety, and direct loneliness affected parental mental health. Parent status, receiving support and giving support, overcrowding, food insecurity, time for one's self prior to COVID-19, and income were included as predictors to examine different influencers on parental mental health (depressive, anxiety, and loneliness) in relation to parental gender, ethnicity, financial insecurity using hierarchical multiple regression analyses (60). In step 1 of the depression regression, parental gender, ethnicity, and current financial insecurity (hence forth referred to as financial insecurity) were entered as control variables. In step 2, predictors were entered in the proceeding order: parental gender, ethnicity, financial insecurity, parent status, giving support and receiving support, overcrowding, food insecurity, time for one's self prior to COVID-19, income, and child access to outdoor space.

In step 1 of the anxiety regression, parental gender, ethnicity, current financial insecurity, food insecurity, and time for self were entered as control variables. In step 2, predictors were entered in the proceeding order: parental gender, ethnicity, current financial insecurity, food insecurity, time for one's self prior to COVID-19, parent status, giving support and receiving support, overcrowding, and income.

In step 1 of the loneliness regression, parental gender, ethnicity, current financial insecurity, parent status, and food insecurity were entered as control variables. In step 2, predictors were entered in the proceeding order: parental gender, ethnicity, current financial insecurity, parent status, food insecurity, time for self, and income.

### Profile of Mental Health of Parents in Tower Hamlets

No depressive symptoms were found for 36% of parents and 29% experienced mild depressive symptoms. Moderate to moderate severe depression was higher in fathers (34%) and mothers (35%) (total sample: 35%) than nationally (19%, ONS, Opinions and Lifestyle Survey July 4–14, 2020, PHQ-8).

No symptoms of anxiety were found for 41% of parents, while 59% experienced symptoms of anxiety (30% mild anxiety, 17% moderate anxiety, and 11% severe anxiety). Similar prevalence of symptoms of anxiety was found for mothers (58%) and fathers (61%) (GAD-7). This is in contrast to national levels (17%, ONS, November 2020; 2 items from GAD-7). Similar prevalence of experiencing direct loneliness was found for mothers (20%) and fathers (22%).

### Income and Financial Insecurity

Parental mental health was not correlated with *changes* in financial security since March 2020 (pre-COVID-19) (*p* > 0.05) (“Compared to before lockdown started in March 2020, how would you say you are doing financially right now?”) and was subsequently dropped from the regression models. Changes in parental financial insecurity in Tower Hamlets, since March 2020, do not influence the mental health of parents in our sample. We report *current* financial insecurity below; “How well would you say you are managing financially right now” (hence forth referred to as financial insecurity).

We were interested in possible facilitatory or preventative roles financial factors played in parental mental health. Financial impacts are cited as one of the main influences on parental mental health (5, 17). Larger cohort studies have found that financial insecurity is a predictor of mental health during COVID-19 (17, 31).

Income and *current* financial insecurity (0 = financially insecure, 1 = financially secure) both significantly correlated with depression (income *r* = −0.23, *p* < 0.001, financial insecurity (*r* = −0.38, *p* < 0.001), and anxiety (income *r* = −0.21, *p* < 0.001, financial insecurity *r* = −0.37, *p* < 0.001). But only feeling financially insecure (0 = financially insecure, 1= financially secure) predicted experiencing depressive symptoms (*B* = −0.74, β = −0.31, *p* < 0.001) and experiencing symptoms of anxiety (*B* = −0.70, β = −0.34, *p* < 0.001). Income was not a significant predictor (*p* > 0.05).

Income and *current* financial insecurity both significantly correlated with loneliness (income *r* = 0.21, *p* < 0.001, financial insecurity *r* = 0.25, *p* < 0.001). Feeling financially insecure predicted experiencing loneliness (*B* = 0.11, β = 0.14, *p* < 0.05). Income was not a significant predictor (*p* > 0.05).

In our study, parents feeling financially insecure were significantly more likely to experience depression symptoms, symptoms of anxiety, and loneliness. This finding mirrors that of the majority of the literature which focuses on parents nationally as well as non-parents using large cohort studies, such as UK Household Longitudinal Study (17, 68, 69). We expected to also find that income predicted mental health problems but speculate parents' feelings of financial insecurity influence parental mental health more than income alone. Indeed, Kopasker et al. (10) found that financial insecurity predicted mental health difficulties irrespective of adults' income. Although, Cheng et al. (17) found that financial insecurity, as a predictor of mental health, was mediated by income type, with lower income groups experiencing greater financial insecurity.

### Food Insecurity

Food insecurity significantly correlated with depression (*r* = 0.302, *p* < 0.01) and symptoms of anxiety (*r* = 0.31, *p* < 0.01). Food insecurity predicted experiencing depressive symptoms (*B* = 0.90, β = 0.23, *p* < 0.001) and experiencing symptoms of anxiety (*B* = 0.90, β = 0.26, *p* < 0.001). Parents who experienced food insecurity were significantly more likely to experience depressive symptoms and symptoms of anxiety compared to parents who were food secure.

This finding is unsurprising, as not knowing whether one can feed one's self and children creates feelings of anxiety and shame (70). Our findings speak to that of previous findings of poor mental health in adults who are food insecure, compared to food secure (47, 70).

Food insecurity significantly correlated with loneliness (*r* = −0.28, *p* < 0.01). Food insecurity predicted experiencing loneliness (*B* = −0.39, β = −0.28, *p* < 0.001). Parents who experienced food insecurity were significantly more likely to experience loneliness compared to parents who were food secure, once all other covariates were taken into account. Our findings speak to previous literature which has found associations between loneliness and food poverty (71). Food insecurity in this group may arise due to a reduction in eating food thus reducing social interactions enjoyed through communal meals (71).

Covariates which significantly correlated with food insecurity were ethnicity (*r* = 0.10, *p* < 0.05) financial insecurity (*r* = −0.23, *p* < 0.01), income (*r* = −0.24, *p* < 0.01), parent status (*r* = 1.11, *p* < 0.01), and time for one's self prior to COVID-19 (*r* = −0.13, *p* < 0.01). Parental food insecurity was associated with parents experiencing financial insecurity, cohabiting parents, those with less time for themselves, and those in low income households. Future analyses can examine mediating roles of time for one's self, financial insecurity, and parent status on food insecurity and parental mental health.

### Parental Time for Self

We were interested in possible influences of changes in parental time for self since March 2020 (the one remaining variable that examines change). Differences in parental time for self, compared to before the March 2020 lockdown, were significantly correlated with experiencing depressive symptoms (*r* = −0.21, *p* < 0.01), symptoms of anxiety (*r* = −0.20, *p* < 0.01), and loneliness (*r* = 0.10, *p* < 0.01). Differences in parental time for self were not a significant predictor for depression (*p* = 0.06) or loneliness (*p* = 0.10). Differences in parental time for self were a significant predictor of anxiety (*B* = −0.20, β = −0.17, *p* < 0.001) once all covariates were controlled for. Covariates which significantly correlated with time for self were income (*r* = −0.02, *p* < 0.01), ethnicity (*r* = −0.25, *p* < 0.01), parental gender (*r* = 0.28, *p* < 0.01), overcrowding (*r* = −0.15, *p* < 0.01), food insecurity (*r* = −0.13, *p* < 0.01), receiving support (*r* = −0.08, *p* < 0.05), parent type (*r* = 0.10, *p* < 0.01), and financial insecurity (*r* = 0.12, *p* < 0.01).

Parents with less time for self, compared to pre-COVID-19, were significantly more likely to have symptoms of anxiety. Our findings speak to evidence of reduction in time for self of parents since COVID-19 (17, 55, 56) and time for self as a possible influence on parental anxiety levels (55).

### Adequate Housing

Although overcrowding was not significantly correlated with depression (*p* = 0.17), anxiety (*p* = 0.19), or loneliness (*p* = 0.39), we considered it to be of great importance to mental health (14, 50) and therefore it was included in the final regression models for depression and anxiety. However, overcrowding did not significantly predict experiencing depressive symptoms or symptoms of anxiety (*p* > 0.05) once all other variables were accounted for. Future analyses could consider mediating factors of food insecurity, financial insecurity, and leisure time (the main significant predictors of mental health in parents) between mental health and overcrowding in parents.

### Parent Status, Quality of Relationships, and Support

Parent status (cohabiting vs. lone) was significantly correlated with depression (*r* = −0.15, *p* < 0.01), symptoms of anxiety (*r* = −0.11, *p* < 0.01), and loneliness (*r* = 0.19, *p* < 0.01). Examining the direction of this relationship, for depression and anxiety there was a significant association between lone parents experiencing more depression and symptoms of anxiety than cohabiting parents. However, parent type did not predict experiencing depressive (*p* > 0.80) symptoms and symptoms of anxiety (*p* = 0.14).

Parent status was significantly correlated with loneliness (*r* = 0.19, *p* < 0.01). Parent status (*B* = 0.23, β = 0.18, *p* < 0.001) predicted experiencing loneliness. Cohabiting parents were more likely to experience less loneliness, compared to lone parents. It is unsurprising that there was a greater likelihood of lone parents experiencing loneliness; previous research has documented that people living alone are at greater risk of experiencing loneliness during COVID-19 (35, 72).

Quality of relationship with spouse was not correlated with depression, anxiety, and loneliness (*p* > 0.05) and was dropped from regression analyses.

Receiving and giving support were correlated with depression (receiving: *r* = 0.10, *p* < 0.01, giving: *r* = 0.09, *p* < 0.05) and symptoms of anxiety (receiving: *r* = 0.10, *p* < 0.01, giving: *r* = 0.09, *p* < 0.05). However, giving (*p* = 0.09) and receiving (*p* > 0.4) support did not predict experiencing depressive symptoms. Receiving support (*B* = 0.20, β = 0.10, *p* < 0.05) predicted experiencing anxiety symptoms but not giving (*p* = 0.50) support (Table 4).

Receiving and giving support were not correlated with loneliness (*p* > 0.1). It is surprising that support was not related to loneliness, previous literature has highlighted the importance of support, or lack of, in feelings of loneliness pre and during COVID-19 (35).

### Child(ren) Having Access to Outdoor Space

Children having access to outdoor space was correlated with depression (*r* = −0.15, *p* < 0.05) but not anxiety (*p* > 0.05) or loneliness (*p* > 0.05) and was dropped from anxiety and loneliness models. Parents whose child(ren) had access to outdoor space were significantly less likely to have depressive symptoms than those who did not (*B* = −0.40, β = −0.13, *p* < 0.05). We expected anxiety to also be related to child(ren) having access to outside space because parents have shown concerns over their child(ren)'s amount of outdoor play/activity over the lockdown period (5). Yet our findings suggest that only depressive symptoms are linked to child(ren) having access to outside space.

### Ethnicity and Parental Gender

Once all predictors were taken into account, ethnicity significantly predicted depression (*B* = −0.32, β =-0.13, *p* < 0.05). White British/Irish parents are less likely to experience depressive symptoms than South Asian parents. Covariates which significantly correlated with ethnicity were income (*r* = −0.42, *p* < 0.01), overcrowding (*r* = 0.35, *p* < 0.01), food insecurity (*r* = 0.10, *p* < 0.05), time for self (*r* = −0.25, *p* < 0.01), quality relationships (*r* = 0.13, *p* < 0.01), giving support to friends and family (*r* = −0.10, *p* < 0.05), and financial insecurity (*r* = −0.35, *p* < 0.01).

Ethnicity significantly predicted experiencing symptoms of anxiety (*B* = −0.19, β = −0.09, *p* < 0.05) once covariates of parental gender, financial insecurity, time for one's self prior to COVID-19, and food insecurity were taken into account. However, ethnicity no longer predicted anxiety in the final regression model.

Once all predictors were taken into account, parental gender significantly predicted depression (*B* = 0.35, β = 0.12, *p* < 0.05) and anxiety (*B* = 0.19, β = 0.09, *p* = 0.04). Fathers were significantly more likely to experience symptoms of depression and symptoms of anxiety than mothers once all covariates were taken into account.

Covariates which significantly correlated with parental gender were overcrowding (*r* = −0.07, *p* < 0.05), time for one's self prior to COVID-19 (*r* = 0.28, *p* < 0.01), receiving support from friends and family (*r* = −0.08, *p* < 0.05), and parental type (lone vs. cohabiting) (*r* = 0.24, *p* < 0.01). Looking at the direction of these correlations, only parental status and time for one's self prior to COVID-19 was associated with fathers, suggesting fathers had more time for themselves compared to before COVID-19 and more fathers were living with their partner. Only 35% of fathers in contrast to 65% of mothers report less time to themselves compared to before COVID-19 (40% of fathers and 17% of mothers report more time to themselves, and 25% of fathers and 18% of mothers report no change in time for themselves). Our findings that parents with less time for themselves, compared to pre-COVID-19, were significantly more likely to have symptoms of anxiety suggest future analyses could consider mediating roles of time for oneself on symptoms of anxiety.

An additional regression model was adopted to explore the influence of time for one's self prior to COVID-19 on anxiety in mothers. Time for one's self significantly predicted symptoms of anxiety in mothers (Table 6), once ethnicity was controlled for, as did parental status (living without a partner). Ethnicity was also not a significant predictor, suggesting that time for one's self prior to COVID-19 and being a lone parent influence mothers' symptoms of anxiety irrespective of ethnic background. Exploring employment in lone mothers we find that 27% of mothers were employed (currently employed, on maternity leave, or self-employed and working) and 73% were self-employed but not working, unemployed, or unemployed and on benefits.

### Exclusion of Fathers in Regression Models That Explore Time for One's Self and Parent Status

Due to the small sample size of fathers (*N* = 169) in our cohort, the influence of time for one's self and parental status was not modeled. Furthermore, prevalence of lone parent status in our sample was skewed toward mothers with 147 being lone parent mothers and 5 being lone parent fathers.

### Discussion of Key Findings

Key influencers in experiencing depressive symptoms were material assets (financial insecurity, food insecurity, and parents' child(ren) having access to outdoor space). Similar predictors emerged in the anxiety regression model with material (financial insecurity and food insecurity), familial (changes in parental time for self since COVID-19), and community assets (receiving support from friends and family outside the home), influencing symptoms of anxiety. Experiencing loneliness was influenced by material assets (financial insecurity and food insecurity), like depression and anxiety. In addition, familial assets (parental status), such as being in a cohabiting relationship, shielded parents from experiencing loneliness. Lastly, fathers were significantly more likely to experience symptoms of depression and anxiety, compared to mothers, once all covariates were accounted for. South Asian parents were significantly more likely to experience symptoms of depression once all covariates were accounted for. Time for one's self and being a lone parent influenced symptoms of anxiety in mothers.

### Changes in Life Since COVID-19

Although *changes* in financial insecurity since March 2020 did not influence parental mental health and was excluded from regression models, the perceived change in parental time for self since before COVID-19 influenced anxiety. Those parents who experienced less time for themselves were more likely to experience more symptoms of anxiety, compared to before March 2020. Other inner city (57) and national (17) findings report a decline in time for one's self due to fear of COVID-19, and time for self as a possible influence on parental anxiety levels (55). Motivation and opportunity to take part in leisure or other “time for self” activities may be possible reasons for a decrease in time for self during COVID-19 (73). Fear of going outside because of the virus is also a factor (57). Mothers who experienced less time for themselves were more likely to experience more symptoms of anxiety, this pattern was not influenced by ethnicity.

The below quotes from two in-depth interviews conducted as part of the longitudinal aspect of the FTH project emphasize the lack of time for self-experienced by parents, perhaps due to lack of opportunity:

“*honestly we don't have time for ourselves because it's for morning … when I wake at 6 o'clock in the morning, sometimes half 5, till 10 o'clock or 11 o'clock [at night] there is no way that I can sit down.” (Mother, Indian respondent)*

Again, the below quote documents fears experienced that may be associated with not going outside and reduced time for self:

*stressful days because we were always under fear of the unknown, in terms of how the virus impacts you and then what happens. You know, can you survive? And therefore, we spent most of the time indoors (Father, Indian respondent)*

Proposing interventions, where an increase in time for self for parents is facilitated, may be a consideration but for many parents, during COVID-19, a reduction in support network and increased demands on time from home schooling and work (17) mean this may not be obtainable. Wave 2 of the FTH project survey will enable us to consider if anxiety levels have changed since COVID-19 and what other roles, apart from time for self, influence changes in mental health. In line with Yerkes et al. (56), we find that lockdown restrictions, imposed to reduce health costs of COVID-19, have brought a reduction in time for one's self which contributes to anxiety in parents in Tower Hamlets.

## Possible Facilitatory and Preventive Factors on Parental Mental Health

### Loneliness

We argue that loneliness is strongly associated with poor material assets, as detailed using regression analyses; strong predictors of loneliness were financial insecurity and food insecurity, and with our finding that loneliness significantly correlated with low income parents, we suggest that those from financially insecure backgrounds are at greater risk of experiencing loneliness (74). Lone parents were significantly more likely to experience loneliness compared to co-habiting parents, highlighting the influence of familial as well as material assets on loneliness. Parents who also had less time for self, compared to pre-COVID-19, were significantly more likely to experience loneliness. However, a reduction in time for one's self is not dependent only on economic deprivation and occurs across low-to-high income groups (75). A reduction in time for one's self as a vehicle for loneliness speaks to this assumption, perhaps because parents who have less time for themselves feel a greater disconnect from their community and a greater sense of isolation.

Findings regarding loneliness should also be treated with caution in light of the inclusion of a direct measure of loneliness only (How often have you felt lonely during the past week?). ONS guidelines recommend using both standardized indirect [the UCLA Three-Item Loneliness Scale (UCLATILS)] and direct loneliness (Direct Measure of Loneliness (DMOL question) scales (66). Due to the size and scope of the survey adopted in the project, it was not possible to include both an indirect and a direct measure of loneliness. Future research in Tower Hamlets could consider both direct and indirect loneliness assessments.

### Depression and Anxiety

In line with other inner city (5, 57) and national studies [(17); anxiety: (72)] examining parental mental health during COVID-19, we find that parents' symptoms of depression and anxiety were influenced by material assets (financial insecurity and food insecurity) (5, 32, 57). What is more, South Asian parents and fathers across ethnic groups were more likely to experience symptoms of depression, and anxiety for fathers only, when controlling for other factors (21, 76). Finally, time for one's self and being a lone parent influenced symptoms of anxiety in mothers.

Higher than national levels of depression and anxiety and material assets of financial and food insecurity influencing mental health difficulties of parents in Tower Hamlets speaks to a community in a high deprivation area (1) facing multiple adversities which results in a greater likelihood of experiencing poorer mental health during COVID-19 (72). Indeed, our finding of higher than national levels of symptoms of depression and anxiety in Tower Hamlets parents speaks to the precarity of mental health when already experiencing financial difficulty. Those who already experienced financial difficulties prior to COVID-19 had greater mental health difficulties with the additional financial strain experienced during COVID-19 (31). Feeling the pinch of financial difficulties before COVID-19 was common in Tower Hamlets. With the highest rates of child poverty in England (43), it is unsurprising that this group of parents' anxiety would be influenced by financial insecurity during lockdown. Furthermore, more adults from minority ethnic groups are likely to experience financial difficulties during COVID (31), and depression rates were higher during COVID-19 for Bangladeshi and Pakistani men compared to White men (21). This latter finding is in keeping with our findings, that South Asian parents experience more symptoms of depression compared to White British/Irish parents.

Fathers were significantly more likely to report more symptoms of depression and anxiety than mothers, when other factors were controlled for. When considering this finding within the context of the additional predictors of depression and anxiety, financial insecurity and food insecurity, fathers who experience financial insecurity and food insecurity may be more likely to experience anxiety perhaps because in our sample, more fathers are employed than mothers (16). Kopasker et al.'s (10) work around the “bread winner hypothesis” suggests that financial insecurity is a stronger predictor of mental health difficulties for men compared to women. Evidence from a recent London-based survey also suggests that financial insecurity is at the forefront of stressors for families living in London (77).

As expected we found that parents experiencing symptoms of anxiety were more likely to receive support than parents not experiencing anxiety. This highlights the necessity of parents' support networks (community assets) when dealing with life during lockdown. Receiving support predicted experiencing symptoms of anxiety but not giving support. Perhaps because more parents who experience symptoms of anxiety receive support from their family and friends than parents who do not experience symptoms of anxiety.

Anxiety levels associated with a reduction in time for self and receiving less support from friends and family members outside of the household highlight the importance of familial and community assets: having time for oneself and access to peer and familial support during a pandemic. Future government planning should consider the effects lockdown restrictions have on parents' mental health and the knock on effects this has on child mental health [(78); USA: (79)].

Parents not having access to outside space for their child(ren) was associated with depression. This, and our earlier finding that parents who have less time for self since March 2020 are significantly more likely to experience symptoms of anxiety, suggests a need to facilitate access to outside space and leisure activities. Tower Hamlets' strategy to increase physical activity and play [(46); Play charter; (80)] to tackle poor mental health, speaks to the importance of our findings. Future analyses will consider these areas using Wave 2 data.

The below quotes, taken from the qualitative part of the FTH project speak to the stresses experienced by parents who cannot take their children out to play/exercise:

*the beginning of lockdown he was sad that he couldn't go into the playground because all the playgrounds were closed. Now the playgrounds are open, but I actually don't think … I'm not really happy with going into the playground because he's 4 and he touches everything. And even though if he were to get Covid he probably would be okay, and I probably would be okay, my mum's living with us and it's just too much risk. (Mother, White British/Irish respondent)*

*Not being able to take my son out or to his play groups has been difficult because we don't have a garden. (Mother, Bangladeshi respondent)*

We further explored the influences on anxiety for mothers and although we cannot infer what distinguishes mental health in fathers and mothers, due to low sample size of fathers, we can infer what influences mothers' anxiety. Anxiety levels in mothers were associated with living in lone parent households, and unsurprisingly having less time for one's self. High prevalence of lone parent mothers in our sample and influence of lone parent status and time for one's self on anxiety relates to overload experienced by lone parents before and during COVID-19 (81). Evidence of the protective quality of employment for lone mothers' mental health (82) speaks to the minority of our lone mother sample being currently in employment (27%).

The struggles of too many demands on one's time during COVID-19 are well-documented (17, 35, 56, 83) as well as difficulty finding childcare (84) which could contribute to anxiety in mothers. Although previous research has included parent type (cohabiting vs. lone parent), little is known about the mental health of lone parents during COVID-19. We find that having less time for one's self and being a lone mother influences symptoms of anxiety in mothers irrespective of ethnicity.

## Limitations

There are several data limitations which should be taken into account in the interpretation of findings. Firstly, there is a low sample size particularly for fathers in both ethnic groups and parental status (no partner) for both mothers and fathers. The low sample size means we cannot ensure our sample is representative and indicative of the perspectives of some ethnic groups in Tower Hamlets or is of sufficient power to compare to other inner city boroughs in mid to high income countries. Although, our choice of examining mental health in White British/Irish and South Asian parents was based on low sample size in other ethnic groups. Secondly, due to missing data, a proportion of mothers and fathers in the main study did not complete all items of standardized anxiety and depression measures so were excluded from regression analysis. Therefore, the full sample was not included in regression analyses.

## Conclusion and Future Considerations

In conclusion, parental anxiety and depression in this study are higher than the national average. Possible preventers or facilitators of good mental health are material assets (financial insecurity, food insecurity, and child(ren) having access to outside space for depression), familial assets (changes in time for self since COVID-19; parent status: lone vs. cohabiting), and community assets (receiving support outside of the household). Suggesting, in line with previous research, material as well as familial assets influence parents as well as their children's mental health (85). These possible preventers are in line with the Tower Hamlets Bounce Back plan (80) which discusses the importance of active lifestyle and support for children in Tower Hamlets as a means of “Bouncing Back” after COVID-19. We know the effects parental mental health has on child mental health (26) and deem our findings to speak to the importance of reducing financial insecurity and food insecurity as a means of improving mental health of parents so that every child has a safe space in which to thrive (46).

## Data Availability Statement

The raw data supporting the conclusions of this article will be made available by the authors, without undue reservation.

## Ethics Statement

The studies involving human participants were reviewed and approved by the UCL Institute of Education Research Ethics Committee (REC1366) and the NHS Health Research Authority (20/LO/1039). The patients/participants provided their written informed consent to participate in this study.

## Author Contributions

HH and KH recruited participants. CC, MO'B, HH, and KH designed and created the survey. LW cleaned, analyzed the quantitative data, and drafted the first version of the manuscript. KH analyzed the qualitative data. LW, CC, and MO'B amended further iterations of the manuscript. All authors contributed to the article and approved the submitted version.

## Funding

The research project on which this paper draws is COVID-19: Families, children aged 0-4 and pregnant women: vulnerabilities resources and recovery in Tower Hamlets and was funded by the UK Economic and Social Research Council (ES/V004891/1).

## Conflict of Interest

The authors declare that the research was conducted in the absence of any commercial or financial relationships that could be construed as a potential conflict of interest.

## Publisher's Note

All claims expressed in this article are solely those of the authors and do not necessarily represent those of their affiliated organizations, or those of the publisher, the editors and the reviewers. Any product that may be evaluated in this article, or claim that may be made by its manufacturer, is not guaranteed or endorsed by the publisher.

## Footnotes

^1^Health Foundation analysis of the English Housing Survey, MHCLG. 2015-2019.

^2^Born in Bradford. (2021). https://borninbradford.nhs.uk/research/.
